# Malaria infection among adults residing in a highly endemic region from the Democratic Republic of the Congo

**DOI:** 10.1186/s12936-024-04881-7

**Published:** 2024-03-18

**Authors:** Nadine Kalenda Kayiba, Yuko Nitahara, Evariste Tshibangu-Kabamba, Denis Kalambayi Mbuyi, Augustin Kabongo-Tshibaka, Nestor Tshituka Kalala, Barthélemy Mukenga Tshiebue, Katherine-Sofia Candray-Medina, Natsuko Kaku, Yu Nakagama, Niko Speybroeck, Dieudonné Ngoyi Mumba, Ghislain Tumba Disashi, Akira Kaneko, Yasutoshi Kido

**Affiliations:** 1 Research Center for Infectious Disease Sciences, Graduate School of Medicine, Osaka Metropolitan University, Osaka, Japan; 2 Departments of Virology and Parasitology, Graduate School of Medicine, Osaka Metropolitan University, Osaka, Japan; 3 Department of Public Health, Faculty of Medicine – Pharmacy and Public Health, University of Mbujimayi, Mbuji Mayi, Democratic Republic of Congo; 4 Research Institute of Health and Society, Université Catholique de Louvain, Brussels, Belgium; 5 Department of Internal Medicine, Faculty of Medicine – Pharmacy and Public Health, University of Mbujimayi, Mbuji Mayi, Democratic Republic of Congo; 6 Department of Molecular Infection Dynamics, Institute of Tropical Medicine, Nagasaki University, Nagasaki, Japan; 7 Department of Parasitology, Institut National de Recherche Biomédicale, Kinshasa, Democratic Republic of Congo; 8 Department of Microbiology, Tumor and Cell Biology, Karolinska Institutet, Stockholm, Sweden

**Keywords:** Malaria, Prevalence, Risk factors, *Plasmodium*, Africa, The Democratic Republic of Congo

## Abstract

**Background:**

Adults infected with *Plasmodium* spp. in endemic areas need to be re-evaluated in light of global malaria elimination goals. They potentially undermine malaria interventions but remain an overlooked aspect of public health strategies.

**Methods:**

This study aimed to estimate the prevalence of *Plasmodium* spp. infections, to identify underlying parasite species, and to assess predicting factors among adults residing in an endemic area from the Democratic Republic of Congo (DRC). A community-based cross-sectional survey in subjects aged 18 years and above was therefore carried out. Study participants were interviewed using a standard questionnaire and tested for *Plasmodium* spp. using a rapid diagnostic test and a nested polymerase chain reaction assay. Logistic regression models were fitted to assess the effect of potential predictive factors for infections with different *Plasmodium* spp.

**Results:**

Overall, 420 adults with an estimated prevalence of *Plasmodium* spp. infections of 60.2% [95% CI 55.5; 64.8] were included. Non-*falciparum* species infected 26.2% [95% CI 22.2; 30.5] of the study population. Among infected participants, three parasite species were identified, including *Plasmodium falciparum* (88.5%), *Plasmodium malariae* (39.9%), and *Plasmodium ovale* (7.5%) but no *Plasmodium vivax*. Mixed species accounted for 42.3% of infections while single-species infections predominated with *P. falciparum* (56.5%) among infected participants. All infected participants were asymptomatic at the time of the survey. Adults belonging to the “most economically disadvantaged” households had increased risks of infections with any *Plasmodium* spp. (adjusted odds ratio, aOR = 2.87 [95% CI 1.66, 20.07]; p < 0.001), compared to those from the "less economically disadvantaged” households. Conversely, each 1 year increase in age reduced the risk of infections with any *Plasmodium* spp. (aOR = 0.99 [95% CI 0.97, 0.99]; p = 0.048). Specifically for non-*falciparum* spp., males had increased risks of infection than females (aOR = 1.83 [95% CI 1.13, 2.96]; p = 0.014).

**Conclusion:**

Adults infected with malaria constitute a potentially important latent reservoir for the transmission of the disease in the study setting. They should specifically be taken into account in public health measures and translational research.

**Supplementary Information:**

The online version contains supplementary material available at 10.1186/s12936-024-04881-7.

## Background

Malaria is a life-threatening illness caused by *Plasmodium* spp. parasites transmitted to humans through the bites of infected mosquitoes. Despite being preventable and treatable, the disease remains a major public health problem globally, with its highest burden occurring in Africa (i.e., ~ 95% of cases and associated deaths) [1]. More than 200 million cases are reported yearly across African countries due to four main *Plasmodium* species, including *Plasmodium falciparum*, *Plasmodium malariae*, *Plasmodium ovale*, and *Plasmodium vivax* [1]. Among them, *P. falciparum* infections are of greatest concern as they are the most prevalent and fatal due to serious complications occurring mainly in children under 5 years old and pregnant women [1]. Beyond its high morbidity and mortality across the continent, malaria diverts substantial financial resources from individuals and countries towards prevention and treatment efforts [2, 3]. Thus, while affecting the health and wealth of nations, it is both a consequence and a cause of poverty and inequalities [3, 4]. To address this situation, the World Health Organization (WHO) launched the “Global Technical Strategy (GTS) for Malaria 2016–2030” as a global agenda to eliminate malaria by 2030, with clear targets and specific milestones [5]. Toward this ambitious plan, the WHO urges countries to implement interventions tailored to local conditions, with a strong emphasis on vector control, early diagnosis, and rapid treatment [1, 5]. However, several African countries have not yet succeeded in achieving GTS milestones despite efforts [6–8]. In these countries, adults exposed to *Plasmodium* spp. are often overshadowed by better-targeted children under five and pregnant women, even though they represent a major challenge for malaria control and elimination efforts. To optimally meet WHO’s ambitions, existing strategies must be optimized, and new strategies developed to account for all population categories including adults infected with *Plasmodium* spp.

Adults infected with *Plasmodium* spp. represent a particularly complex problem for the epidemiology and pathogenicity of malaria in high endemicity settings such as Sub-Saharan Africa [9]. Indeed, cumulative exposure to *Plasmodium* spp. leads to the acquisition of anti-parasitic and anti-disease immunity that reduces blood parasite levels, prevents symptoms, and provides substantial protection against severe malaria and associated deaths [10, 11]. Adults logically accumulate more infections over time and almost all develop this immunity, unless they have specific underlying health problems such as pregnancy or HIV/AIDS [10]. However, the acquired immunity remains partial and does not fully prevent infections. Semi-immune adults can still be infected with *Plasmodium* spp. while developing mild or no symptoms and carrying parasites in their blood for prolonged periods [11–13]. In sub-Saharan Africa, community surveys report *Plasmodium* spp. prevalence estimates above 70% but also below 5% among asymptomatic individuals [14–19]. Regional variations are likely linked to several local factors, including broad ecological variations (e.g., altitude, temperature, seasons), different existing vector species, and varying intervention coverage. Although often asymptomatic, chronic infections with *Plasmodium* spp. may have a long-term impact on the health of individuals with complications such as recurrent symptomatic episodes, anemia, and cognitive impairment [12, 15]. Ultimately, they would substantially contribute to lower productivity, higher healthcare costs, and increased economic strain among African populations already facing other societal issues. Moreover, infected adults often carry low blood levels of parasites in endemic areas thereby posing significant challenges to standard diagnostic approaches, including rapid diagnostic tests (RDTs) and conventional microscopy [8, 12, 13]. As the expertise and diagnostic tools needed to properly address these challenges are still very limited on the continent, eliminating malaria is proving particularly difficult. Since asymptomatic adults do not necessarily seek care, they would persist in the community while retaining their ability to transmit *Plasmodium* to others through mosquito bites. They thereby constitute an important part of the living reservoir of parasites which maintains infections within communities [11–13, 20–23]. *Plasmodium* spp. infections in adults therefore affect both individuals and the wider community, requiring further recognition through policy decision-making and tailored interventions in the light of targeted GTS milestones.

Located within the WHO African region, the Democratic Republic of Congo (DRC) is one of the countries where adults infected with *Plasmodium* spp. represent the most a gap in public health. This vast country has continuously hosted more than 10% of the global malaria burden, especially since 2015 [1, 6, 24, 25]. Nearly 97% of the Congolese population resides in areas where malaria transmission is stable 8–12 months per year, involving *P. falciparum*, *P. malariae*, *P. vivax*, and *P. ovale* [19, 26–29]. A significant proportion of community infections with these *Plasmodium* spp. is, therefore, expected among semi-immunized adults [25]. Moreover, beyond the country's tropical climate and inadequate sanitation, socio-economic factors such as poverty and lack of education likely make more adults vulnerable to malaria [4]. However, public health efforts aimed at mitigating the impact of malaria have preferentially focused on population categories other than adults, notably young children and pregnant women [13, 25]. Likewise, malaria diagnosis and treatment policies remain primarily focused on *P. falciparum* [25, 30] despite that three other *Plasmodium* spp. (i.e., *P. malaria*, *P. ovale*, and *P. vivax*) are endemic across the country and would be more prevalent among adults. Furthermore, in the absence of targeted interventions, adults infected with *Plasmodium* spp. remain underreported in national surveys such as the Multiple Indicator Cluster Surveys (MICS), the Health Management Information System (HMIS), or the District Health Information Software 2 (DHIS). The prevalence and impact of *Plasmodium* spp. infections are thus widely underestimated in adults. Although *P. falciparum* remains the major species, non-*falciparum* species may increasingly contribute to infections [11, 22]. They could therefore raise additional obstacles to malaria elimination efforts due to limited access to accurate diagnosis, to possible divergent responses to anti-malarial drugs, and to the presence of dormant liver-stage parasites associated with relapses [11]. In this context, the country’s aim to reduce its malaria-related morbidity and mortality by 90% by 2030 [25], could be difficult to achieve without an adequate response to adults infected with *Plasmodium* spp. Further research on *Plasmodium* spp. is warranted to draw deserved attention to infections among adults and provide evidence for tailored interventions to achieve sustainable malaria control throughout the country. The presents study was therefore conducted in an endemic area of the DRC to estimate the prevalence of *Plasmodium* spp. infections among adults while determining the underlying parasite species, associated clinical aspects and predictive factors. This work is timely and relevant to national malaria programmes in the DRC and the most endemic regions of SSA, where the GTS elimination milestones appear most difficult to achieve and sustain.

## Methods

### Study area

**Fig. 1:**
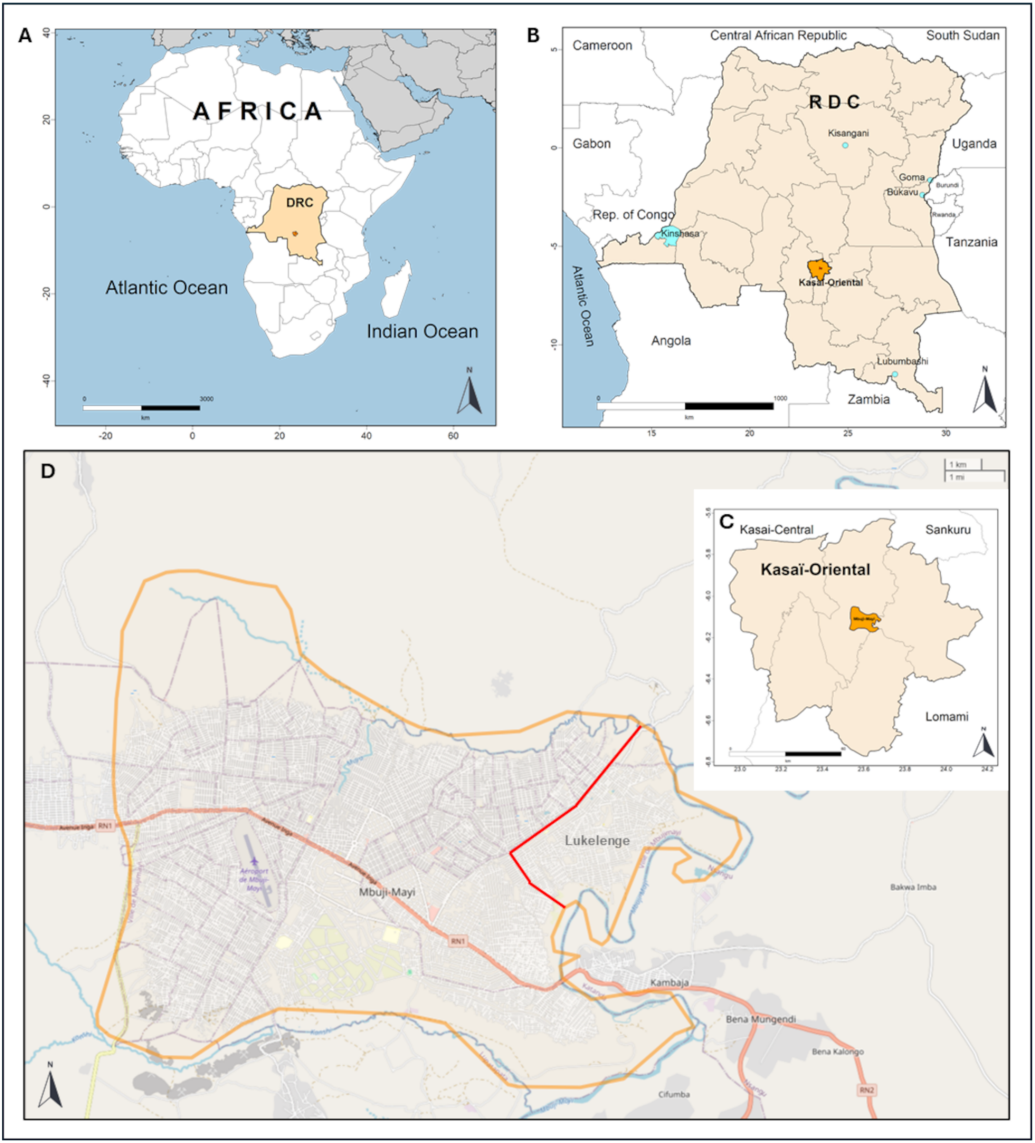
The study sites. This figure displays at different geographical scales the location of the Lukelenge health district which served as a study site, within Africa (**A**), the Democratic Republic of Congo—DRC (**B**), the province of Kasaï-Oriental (**C**), and Mbujimayi city (**D**)

This study was carried out in the Lukelenge health zone which is located at approximately 533 m altitude and extends between − 6.3 and − 6.1 latitude and 23.61 to 25.63 longitude, at the western outskirts of Mbujimayi (Fig. 1). Mbujimayi is the capital city of Kasai Oriental—a region of 9425 km^2^ corresponding to the smallest province of the DRC. The land use pattern in this region is heterogeneous, with densely populated areas separated by large semi-rural and rural areas where agriculture is practiced. Administratively, Kasai Oriental comprises one city (i.e., Mbujimayi) surrounded by five rural territories (i.e., Tshilenge, Katanda, Kabeya Kamuanga, Miabi, and Lupatapata). Two thirds of the region’s population (3,189,886 inhabitants) reside in Mbujimayi with a density of 326 inhabitants per km^2^ [31]. The local health system is organized as a health district, divided into 18 health zones. Health zones are the primary operational units that autonomously plan and implement national health policies, each covering an area of 100,000 to 150,000 and 200,000 to 250,000 inhabitants in rural and urban areas respectively [26, 32]. On a national scale, the DRC’s health system has a pyramidal architecture with the health zone as its base. Divided on average into 15 health areas which cover 10,000 to 15,000 inhabitants, a health zone includes a general referral hospital connected to a network of health centers and community health workers interacting with the population [32]. Mbujimayi city spans nine health zones, including Lukelenge which has a semi-rural profile. Predictions from the Malaria Atlas Project (MAP) (https://malariaatlas.org/pf-pv-cubes-2019/) [5, 7] locate Lukelenge in an area of significant endemic malaria within the country (Additional file 1: Fig S1). The climate is tropical and humid (AW4 according to the Koppen classification), with a long rainy season lasting from September to April and a short dry season, from May to August [33]. The present field survey took place in August 2018, at the end of the dry season.

### Study population and design

A cross-sectional community-based survey at the level of households in the study area was carried out. Households were selected using a simple random sampling process based on a frame established from satellite images and geospatial datasets that were captured over the study area with Google Earth (www.google.com/intl/eng/earth). In each household, a single adult (i.e. an individual aged ≥ 18 years) was recruited according to the “first come, first included” principle. Among potentially eligible participants, those for whom a good quality blood sample or complete interview could not be obtained were excluded. The minimum sample size was determined by assuming that this was a single population survey with a 33.8% prevalence of *Plasmodium* spp. infections, based on existing estimates [28] and a marginal error of 5%, a confidence interval of 95% (z-score = 1.96), and an expected coverage rate of 90%. Therefore, while the survey targeted a minimum of 378 subjects, 420 were ultimately included, resulting a statistical power above 90%.

### Data collection and definition of variables

A standard semi-open questionnaire was used to collect information about participants and their households through an individual interview and brief clinical examination (Additional file 2). In addition to sociodemographic data, individual information collected included use of any malaria preventive measures, possible uptake of anti-malarial drugs, and history of putative symptomatic malaria episodes in the last 3 days, 1 month, and 6 months. Additionally, during the survey, axillary temperature and test results for *Plasmodium* spp. on each participant were recorded. Subsequently, a symptomatic malaria episode was defined based on detected fever (i.e., axillary temperature above 37.7 °C) or on a self-reported history of acute symptoms, including fever, chills, generalized body aches, or headache. Conversely, during the survey, an asymptomatic *Plasmodium* spp. infection was defined based on as a positive polymerase chain reaction (PCR) result for *Plasmodium* spp. without any prior antimalarial drugs uptake and any malaria acute symptoms occurring over the last 3 days. Furthermore, different study variables were combined to define different categories of participants. Participants were considered to have a “high” or “low” education level whenever they had or had not reached secondary school. Besides individual information, the questionnaire also covered the characteristics of the households to which each study participant belonged. Housing conditions (i.e. the structure of walls, floors, and roofs of houses) were used to define a two-level socio-economic categorization of households based on principal components analysis (PCA). As a result, households were classified as being among either “most economically disadvantaged” or “least economically disadvantaged”. Finally, the number of household members sharing a sleeping room was used to define an occupancy index reflecting the crowding of houses. “Overcrowded households” were households whose occupancy index was higher than that of the median population index.

### Blood samples processing and malaria testing

A skin puncture on a participant's finger was performed to collect capillary blood samples for malaria testing. A fresh drop of blood was thus gently transferred to a SD Bioline Malaria Ag Pf test (Standard Diagnostics, Suwon city, Republic of Korea) for detecting the histidine-rich protein 2 (HRP2) of *P. falciparum* according to the Manufacturer’s instructions. Then, 70 µl of blood were sampled with a single-use 75 mm micro-hematocrit capillary tube (Thermo Fisher Scientific, MA, USA). Each blood sample was spotted and dried at ambient temperature on a Whatman 903 filter card (GE Healthcare Ltd., UK) for storage in a zipped plastic bag including a desiccant within it. Dried blood spots (DBS) were afterward shipped to the Department of Parasitology, Osaka Metropolitan University Graduate School of Medicine for molecular testing of *Plasmodium* spp. infections following a previously developed approach [34]. Briefly, a quarter of each DBS (approximately 17.5 µl) was sterilely cut and allocated for DNA extraction using the QIAamp Blood Mini Kit (Qiagen, Hilden, Germany) according to the manufacturer’s recommendations. Then, the mitochondrial cytochrome c oxidase III (*cox3*) gene of *Plasmodium* spp. was amplified using a nested PCR protocol that detects and differentiates the four major *Plasmodium* species (i.e., *P. falciparum*, *P. vivax*, *P. ovale*, and *P. malariae*) [34–36].

### Data analysis

The collected data was entered on a Microsoft Excel spreadsheet (Microsoft Corp., Redmond, WA, USA, 2010) and analysed using R software version 4.1.3 (The R Development Core Team, R Foundation for Statistical Computing, Vienna, Austria). Therefore, several R packages including *tidyverse* [37], *malariaAtlas* [38], *eulerr* [39], *fmsb* [40], *FactoMineR *[41], *Factoextra* *R* [42], and *prevalence* [43] were used for data manipulations, statistical analyses, and graphical representations. The agreement between diagnostic methods was thus qualitatively assessed using the Cohen's kappa statistics and its confidence intervals followed by null-hypothesis testing (i.e., the extent to which the agreement is random or the kappa statistic equals zero) [44]. The Kappa statistic was then interpreted as indicated in Table 1 [45]. A PCA was conducted for defining socioeconomic categories of participants. The study variables between *Plasmodium* spp. infected and uninfected subjects were compared. Hence, with respect to qualitative and quantitative variables, a Chi-square or a Fisher’s exact test and a Wilcoxon’s rank sum test with continuity correction was used where appropriate. The non-parametric conditions were confirmed through Shapiro–Wilk’s and Levene’s tests. The proportion of each *Plasmodium* species was inferred by estimating Jeffreys’ approximate Bayesian confidence intervals [46]. Finally, univariate and multivariate logistic regression models were fitted to assess the association between different factors and infections with *Plasmodium* spp. Multivariate regression models were assessed using forward and backward approaches to select the final model based on the lowest Akaike Information Criterion (AIC) values. At the end, reference to a threshold of p-value < 0.05 was used to define the significance of all statistical tests.

**Table 1:** Interpretation of the Kappa statistics [45]

Kappa values	Level of agreement	Quality of the agreement
≤ 0.2	No agreement	Inadequate
0.21–0.39	Minimal	
0.40–0.59	Weak	
0.60–0.79	Moderate	Adequate
0.80–0.90	Strong	
> 0.90	Almost perfect	

## Results

### Characteristics of study participants and households

A total of 420 out of 425 participants were included in the survey, with a response rate of 98.8%.

Participants had a median age of 29 years (interquartile range, IQR: 25) and included 60.0% female subjects (n = 252). Female subjects were median 29 years old (IQR: 23.0) and males, 28 years old (IQR: 29.5). Most participants were married (n = 291; 69.2%), practiced Christianity as a religion (n = 405; 96.4%), and had a ‘higher’ education level (n = 325; 77.4%). They mainly belonged to the ‘most economically disadvantaged’ households (n = 281; 66.9%) and had resided in the study area for a median duration of 4 years (IQR: 11.0) (Additional file 1: Table S1). More than one in two participants admitted not having a long-lasting insecticide net (LLIN) to control disease vectors in their respective homes (n = 240; 57.1%). Most of them therefore declared that they had not spent any night under an LLIN during the last night (n = 256; 60.9%). Over a 1 month period, excluding a very small number (n = 7; 1.7%), households would not have used indoor insecticide spraying (Additional file 1: Table S2).

### Prevalence of infections with *Plasmodium* spp. and species composition among asymptomatic adults

**Fig. 2:**
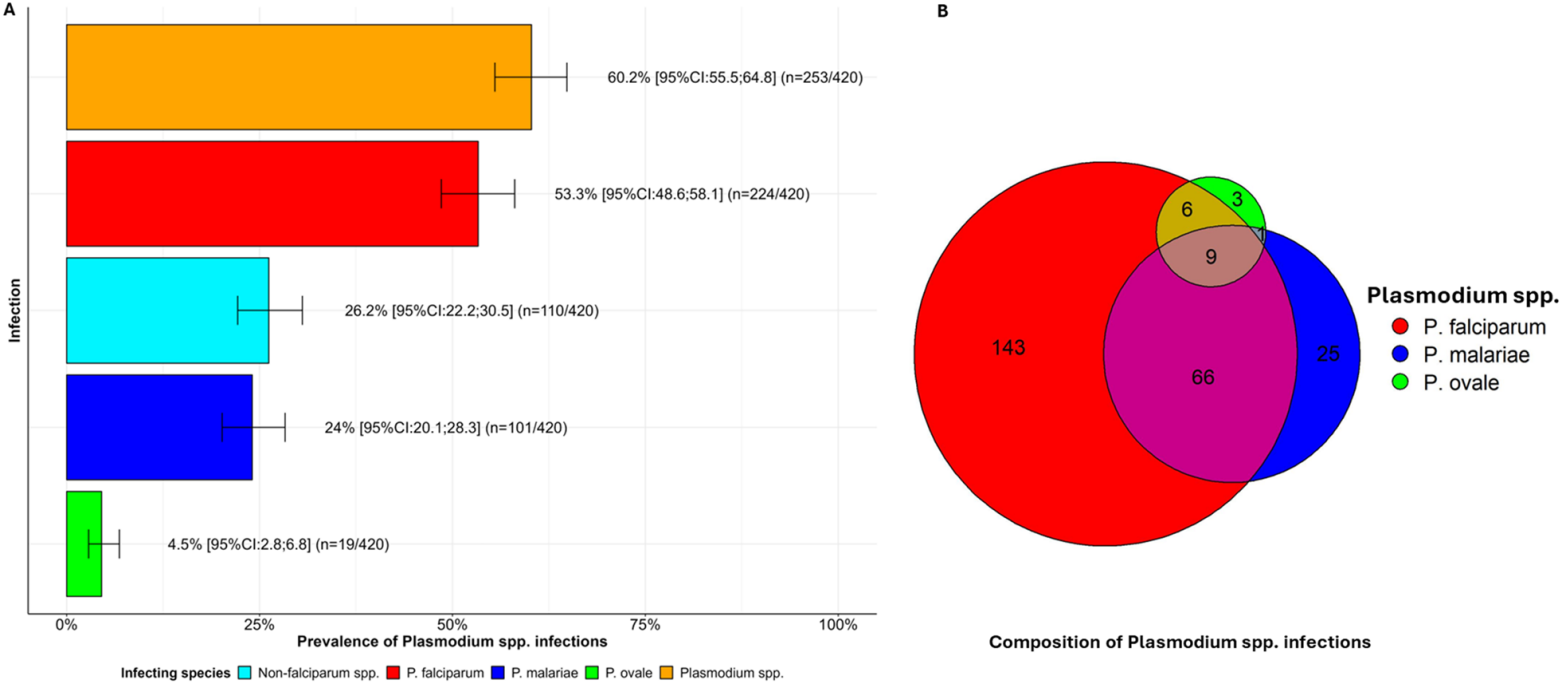
*Plasmodium* species composition and prevalence in the study population. (**A**) Estimated all-age prevalence of *Plasmodium* spp. infections in the study population. This panel displays a histogram of the prevalence of infections with *Plasmodium* spp. species in the study population. Error bars shows 95% confidence intervals (95% CI) estimated as Jeffreys approximate Bayesian confidence intervals around proportions of observed species. (**B**) Schematic representation of the number of *Plasmodium* spp. infections throughout the study population. This panel is a schematic representation of the number of infections with *Plasmodium* spp*.* detected throughout the surveys (total number of tested participants n = 420). The circles indicate number of participants positive for each *Plasmodium* spp. species. The sections where circles overlap represent the number mixed infections with corresponding combinations of more than one *Plasmodium* spp. Colours reflect infecting *Plasmodium* spp. including *P. falciparum* (red), *P. malariae* (blue), and *P. ovale* (green)

**Fig. 3:**
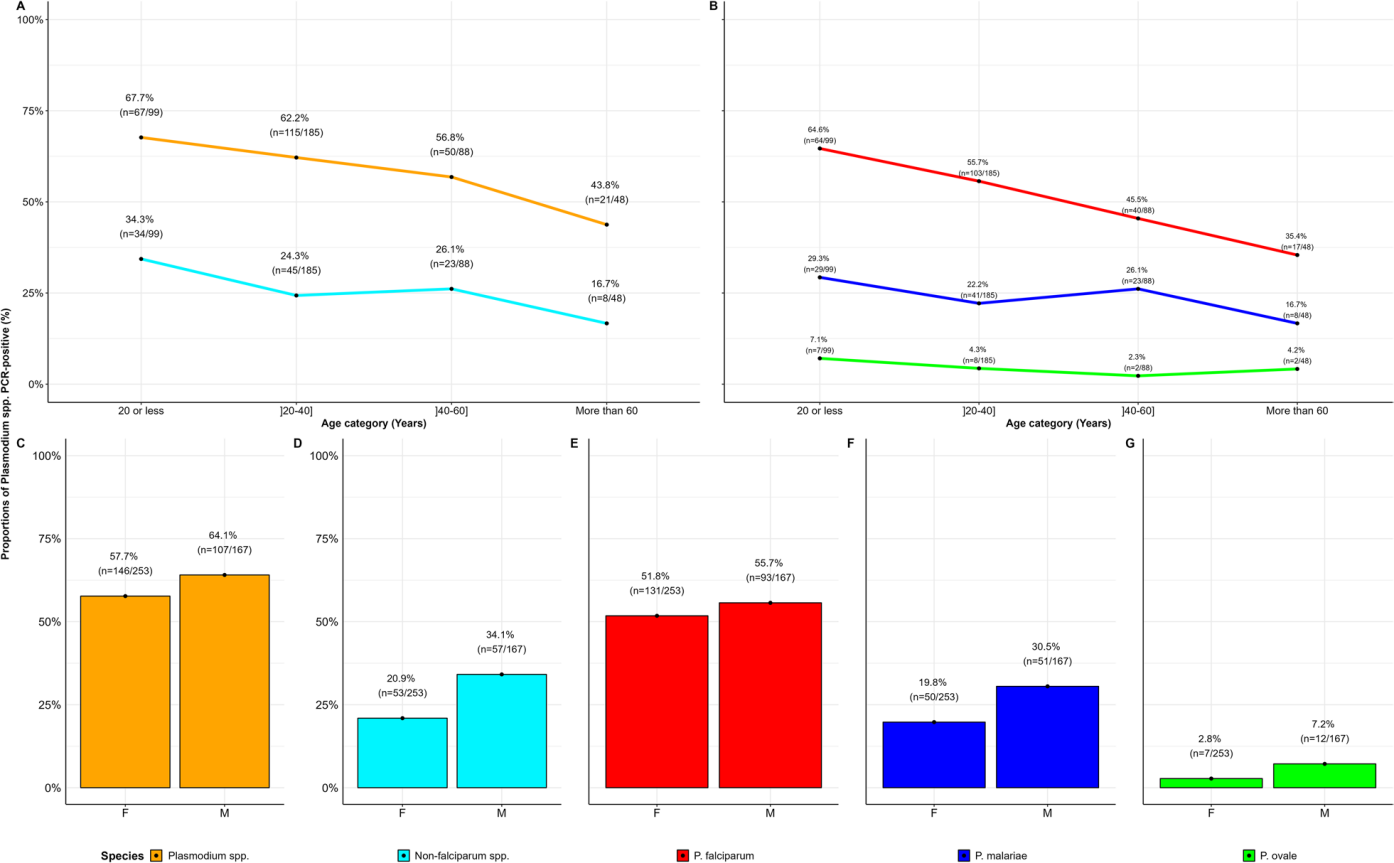
Species-specific stratification by age and gender of *Plasmodium* spp. infected participants. Panels (**A**, **B**) display the proportions observed by age categories, for infections with different *Plasmodium* spp. Panels (**C**–**G**) represent proportions observed by gender categories, for infections with different *Plasmodium* spp

Overall, *Plasmodium* spp. were detected by nested PCR in 253 out of 420 participants and yielded an estimated 60.2% [95% CI 55.5; 64.8] prevalence of infections (Fig. 2). Half of participants reported having experienced at least three potential episodes of symptomatic malaria (IQR: 3) occurring within a 6 month period, including one episode (IQR: 2, 5) dating back less than a month. However, all infected individuals were asymptomatic at the time of the survey (Additional file 1: Table S2). Three *Plasmodium* spp. were identified among infected participants, notably *P. falciparum* as the most prevalent species (53.3% [95% CI 48.6; 58.1]). The prevalence of infections with non-*falciparum* species was estimated 26.2% [95% CI 22.2; 30.5], including *P. malariae* (24.0% [95% CI 20.1; 28.3]) and *P. ovale* (4.5% [95% CI 2.8; 6.8]) but no *P. vivax* infections. *P. falciparum* accounted for 88.5% of infections (n = 224) and non-*falciparum* species accounted for 47.4% (n = 120) of them. Single-species infections thus predominated with *P. falciparum* (n = 143; 56.5%) but existed also with *P. malariae* (n = 25; 9.9%) and *P. ovale (n* = *3; 1.2%).* Mixed *Plasmodium spp.* species were frequent (n = 101; 42.3%) and comprised mainly *P. falciparum—P. malariae* double infections (n = 66; 26.1%) (Fig. 2)*.* Species-specific stratification of infected individuals showed that *Plasmodium* spp. infections decreased with age and slightly predominated in males compared to females (Fig. 3).

### Detection of *Plasmodium* spp. species infecting asymptomatic adults in the population studied

With respect to testing methods, a quarter of RDT-positive subjects (n = 35; 24.5%) tested negative by PCR for *Plasmodium* spp. Oppositely, half of RDT-negative subjects tested positive by PCR for *Plasmodium* spp. (n = 134; 51.3%). Therefore, the results for *Plasmodium* spp. obtained by PCR and HRP-2 based RDT showed minimal agreement (kappa = 0.209 [95% CI 0.118, 0.300]; p < 0.001) (Table 2). Likewise, the tests showed a minimal or no agreement for detecting the *P. falciparum* species (kappa = 0.269 [95% CI 0.176, 0.362]; p < 0.001) or any non-*falciparum* species (kappa = 0.128 [0.018; 0.237]; p = 0.013) (Additional file 1: Tables S3 and S4).

**Table 2:** Qualitative agreement between HRP-2-based RDT and nested PCR assays for *Plasmodium* spp. in the study population (n = 420)

Detection methods*		COX3 nested PCR for *Plasmodium* spp.		Kappa [95% CI]	p-value
		Positive	Negative		
		n (%)	n (%)		
RDT	Positive	108 (75.5)	35 (24.5)	0.209 [0.118; 0.300]	< 0.001
	Negative	134 (51.3)	127 (48.7)		
Total		**242 (59.9)**	**162 (40.1)**		

(*)Additional 11 PCR-positive and 5 PCR-negative participants who had an invalid RDT result are not shown in this table

### Factors associated with *Plasmodium* spp. infections in the study population

Individuals infected with *Plasmodium* spp. had similar characteristics to uninfected ones, with a few exceptions. Infected individuals were a priori younger (26 (IQR: 23) versus 33 (IQR: 31) years; p = 0.002) and belonged more to the “most economically disadvantaged” households (76.1% versus 52.7%; p < 0.001) as well as to those not reporting indoor insecticide spraying over a 1 month period (0.4% versus 3.6%; p = 0.016) (Additional file 1: Tables S3 and S4). Multivariate logistic regression modeling revealed that, with respect to different parasite species, the infection risk was independently influenced by the age and gender of the study participants, as well as the economic level of their households. Therefore, adults belonging to the “most economically disadvantaged” households had an almost three times higher risk of infection by any *Plasmodium* species (adjusted odds ratio, aOR = 2.87 [95% CI 1.66, 20.07]; p < 0.001) or by a *P. falciparum* (aOR = 2.87 [95% CI 1.60, 20.03]; p < 0.001), compared to those from the “less economically disadvantaged” households. Adults from the “most economically disadvantaged” households even had a 5 times higher risk of non-falciparum infections (aOR = 4.87 [95% CI 3.01; 8.08]; p < 0.001). In addition, males had twice the risk of infection with non-*falciparum* species than females (aOR = 1.83 [95% CI 1.13, 2.96]; p = 0.014). Conversely, each one-year increase in age reduced the probability of infection by 1% (aOR = 0.99 [95% CI 0.97, 0.99]; p = 0.048) with respect to the *P. falciparum* species or 2% for all *Plasmodium* spp. combined (aOR = 0.98 [95% CI 0.95; 0.99]; p = 0.048) (Table 3).

**Table 3:** Multivariate logistic regression models identifying variables associated with *Plasmodium* spp. infections in the study population*

Predictive factors and type of infections	Initial model		Final model	
	cOR [95% CI]	p-value	aOR [95% CI]	p-value
Predictive factors of *Plasmodium* spp. infections				
Age (in years)	0.98 [0.97; 0.99]	0.008	0.98 [0.95; 0.99]	0.048
Married, widower/widow, or divorced status	0.63 [0.40; 0.98]	0.023	–	–
Belonging to the ‘most economically disadvantaged’ households	2.81 [1.86; 14.01]	< 0.001	2.87 [1.66; 20.07]	< 0.001
Frequency of putative clinical malaria episodes within the last 6 months	0.94 [0.87; 1.02]	0.124	–	–
No indoor insecticide spraying within the last month	9.39 [1.58; 178.23]	0.039	–	–
Predictive factors of *P. falciparum* infections				
Age (in years)	0.98 [0.97; 0.99]	0.008	0.99 [0.97; 0.99]	0.048
Belonging to ‘Most economically disadvantaged’ households	2.90 [1.88; 17.81]	< 0.001	2.87 [1.60; 20.03]	< 0.001
Frequency of putative clinical malaria episodes within the last 6 months	0.91 [0.84; 0.99]	0.020	–	–
No indoor insecticide spraying within the last month	7.04 [1.19; 133.62]	0.040	–	–
Predictive factors of infections with non*-falciparum* species				
Age (in years)	0.98 [0.97; 1.00]	0.085	–	–
Male gender	1.95 [1.26; 3.04]	0.003	1.83 [1.13; 2.96]	0.014
Married, widower/widow, or divorced status	0.61 [0.38; 0.97]	0.036	–	–
Higher education level	1.84 [1.05; 3.37]	0.039	1.58 [0.87; 3.01]	0.150
Belonging to the ‘most economically disadvantaged’ households	4.83 [3.00; 7.97]	< 0.001	4.87 [3.01; 8.08]	< 0.001

(*) cOR [95% CI]: crude odds ratio with corresponding 95% confidence intervals; aOR [95% CI]: adjusted odds ratio with corresponding 95% confidence intervals

## Discussion

In this study, infections with *Plasmodium* spp. were assessed in adults residing in the Lukelenge health district, a previously unexplored area located in the DRC—one of the two most malaria endemic countries in the world [6]. Overall, adults at the community level, although remaining asymptomatic, were found to be heavily infected with *Plasmodium* spp. and involved a significant contribution from non-*falciparum* species. The socio-economic factors associated with *Plasmodium* spp. infections and the poor diagnostic performance of standard RDTs updated in this work could help design more effective public health measures. Although useful in drawing attention to adults infected with *Plasmodium* spp., our observations add another layer of complexity to the efforts needed to pursue sustainable health goals toward malaria elimination in highly endemic settings.

### Asymptomatic adults are heavily infected with *Plasmodium* spp

A high prevalence (~ 60%) of *Plasmodium* spp. infections was observed among adults. Despite the potentially fulminant nature of *Plasmodium* spp. infections, all infected people reported no symptoms at the time of the survey. This likely reflects a high level of acquired anti-disease immunity in the population [11, 13]. Anti-disease immunity is fundamentally antigen-specific and mediated by genetically distinct clones of parasites providing partial protection against clinical infections [11]. In high-transmission regions like the DRC, residents could be exposed to a diversity of clones, leading to a rapid development of anti-disease immunity and asymptomatic infections [11]. In the literature, the prevalence of *Plasmodium* spp. infections has ranged from less than 5 to more than 70% among asymptomatic adults in various endemic regions of Africa, Southeast Asia, and Latin America [14, 16–18, 22]. More specifically in the DRC, recent surveys including asymptomatic adults have mainly focused on the detection of a single or part of the *Plasmodium* species [19, 27, 28, 47, 48]. Restricting surveys to selected species would have underestimated *Plasmodium* spp. infections. Nevertheless, national surveys have consistently demonstrated a high level of spatial heterogeneity, highlighting the need for subnational investigations and targeted strategies [19, 27]. Indeed, variations in the proportions of asymptomatic infected individuals may result from the levels of *Plasmodium* endemicity and acquisition of anti-disease immunity in each region [11]. However, several other factors would partially explain these variations, notably the species sought, the structure of the population (e.g. age distribution), time-related dynamics (e.g. seasonality), diagnostic methods, and implemented public health strategies.

*Plasmodium* spp. infections among adults from the study area, as probably in most endemic areas, could be of significant concern due to their widespread asymptomatic occurrence and their negative implications for individuals’ health as well as for malaria control and elimination efforts. In fact, infected adults would persist untreated for long periods while being capable to transmit *Plasmodium* spp. to others through mosquito bites, thereby perpetuating the cycle of infections and providing a silent reservoir of parasites within communities [11, 12, 20–23]. In addition, even in the absence of initial symptoms, a long-term and chronic infection with *Plasmodium* spp. can have health consequences such as recurrent episodes of symptomatic parasitaemia, chronic anaemia, maternal and neonatal mortality during pregnancy, cognitive impairment, and co-infection with invasive bacterial infections [12, 15]. This highlights the need to allocate new interventions and adequate resources to specifically address asymptomatic infections among adults in the DRC, a country where diagnosis and treatment primarily trigger individuals with symptomatic infections actively seeking care for clinical illness [24]. Logically, controlling asymptomatic carriage of *Plasmodium* spp. in such an endemic region would raise significant operational challenges of a financial, pragmatic, and political nature; but it is probably a necessary condition for the country to make substantial progress towards malaria control and elimination.

### Non-*falciparum* species contribute significantly to asymptomatic malaria infections among adults and deserve increased attention

The four main *Plasmodium* spp. (i.e., *P. falciparum*, *P. vivax*, *P. malariae*, and *P. ovale*) are known to circulate across the DRC’s ecosystem. Prevalence estimates have thus varied from ~ 7 to ~ 65% for *P. falciparum* [19, 27–29], from 9 to 12% for *P. malariae* [47], from 0 to ~ 5% for *P. ovale* [29, 47, 49], and 0 to ~ 3% for *P. vivax* [29, 48] among adults from different areas. Unlike *P. falciparum* which infected 53.3% of participants and *P. vivax* which was not detected, *P. malariae* (24.0%) and *P. ovale* (4.5%) were more frequent for Congolese settings. While a growing body of evidence has documented *P. vivax* across the DRC and most of Sub-Saharan Africa, the endemicity of this species is expected low at the study site as the Congolese population harbors a Duffy-negative phenotype which confers a natural resistance to this species [48, 50, 51]. When assessing the relative contribution of each species to *Plasmodium* infections, almost 90% of *Plasmodium* spp. involved *P. falciparum* parasites. This is consistent with national epidemiological predictions which could have reasonably encouraged the National Malaria Control Program (NMCP) to focus its diagnostic strategies, therapeutic recommendations, and surveillance activities on *P. falciparum* [25, 52]. However, surprisingly, *P. malariae* and *P. ovale* accounted for almost 43% of *Plasmodium* spp. infections leading to significant mixed (e.g., nearly 36% of *P. falciparum* infections) and single non-*falciparum* species infections (~ 7% of participants). These findings are interesting as they have not been captured in recent surveys among adults where only a few selected *Plasmodium* spp. have been targeted at a time [19, 27–29, 47–49]. Conversely, existing surveys on parasite species composition among asymptomatic children reported very limited numbers of mixed infections and almost no infections with a single non-*falciparum* species [47, 52, 53]. Non-*falciparum* species would be spread diffusely in asymptomatic adults (compared to young children) across the DRC, which may be associated with long-term parasite carriage. Considering the regional vector capacity, all *Plasmodium* spp. detected could be transmitted locally by common vectors that exist across the DRC, namely *Anopheles gambiae* and *Anopheles funestus* [1]. Further research is nevertheless needed to explore possible sources of these infections, as they could still be imported during travel or forced population displacements occurring frequently in the study region [52, 54]. Genotyping parasite populations would provide insights into the genetic diversification of strains, reflecting their relatedness and possible origins [48]. Non-*falciparum* infections therefore increased attention given the potential complexities they may pose for malaria control, management, and research. First, the biology of non-*falciparum* species remains less understood, negatively impacting the successful development of species-specific control strategies [55]. Typically, non-*falciparum* infections may be chronic or dormant for long periods due to hypnozoite formation [55], further complicating the practical understanding of their epidemiology. Second, during co-infections, *P. malariae* can increase *P. falciparum* gametocyte production, suggesting that complex epidemiological interactions exist between species [56, 57]. Third, in addition to their propensity for hypnozoite formation and their differential drug susceptibility, non-*falciparum* species pose significant obstacles to early and accurate diagnosis that potentially undermine any rational management especially in resource-limited settings such as the DRC [55, 58]. Finally, although considered benign, non-*falciparum* infections can cause significant morbidity and overt complications such as anemia [59, 60]. The relevance of non-*falciparum* species is thus increasingly recognized across Africa, particularly when considering malaria elimination goals [54]. Furthermore, a significant contribution of non-*falciparum* isolates to *Plasmodium* spp. infections among adults was observed, highlighting the need for the NMCP to update national policies and practices to target all species appropriately.

### Potent testing tools should be considered to efficiently address *Plasmodium* spp. infections among adults

Conventional microscopy could not be applied to detect and identify *Plasmodium* spp. in this series. This would have required highly qualified and well-trained microscopists, who were lacking on the site at the time of this study, as it is often observed in low-resource settings [55, 61]. Instead, a locally enforced HRP2-based RDT in accordance with national policies was used [25]. RDTs are lateral-flow immunochromatographic devices that had been developed to overcome limitations of conventional microscopy [61]. Unlike microscopy, results obtained with RDTs vary less between examiners and remain as consistent in population surveys as in routine testing, without requiring advanced technical training [61, 62]. Currently available RDTs can achieve a sensitivity that is similar to that of good field microscopy—that is, ∼100 parasites per μl of blood in approximately 5 μl of whole blood [62]. Obviously, microscopy in research settings or reference laboratories may provide greater sensitivity than field microscopy for detecting *Plasmodium* spp.; nevertheless, its detection threshold generally remains > 10 parasites per µl of blood [62]. Meanwhile, infections with *Plasmodium* spp. commonly exhibit very low parasitaemias among asymptomatic adults in endemic areas and raise significant diagnostic challenges, especially when involving non-*falciparum* species [55, 58]. Mixed infections may be masked by more visible concomitant *P. falciparum* and would go unnoticed under microscopy and RDTs [13, 55, 58]. To circumvent these potential diagnostic limitations, an established nested PCR that can detect the four major *Plasmodium* spp. (i.e., *P. falciparum*, *P. malariae*, *P. ovale,* and *P. vivax*) with high sensitivity and specificity was remotely applied [34]. Subsequently, regardless of the species detected, an inadequate agreement was observed between HRP-2-based RDT and nested PCR. This likely reflects a low sensitivity due to high detection thresholds of the RDT used, particularly since nested PCR detected almost twice as many infections, including for *P. falciparum*. Marginal false-negative results with RDT could be due to a functional deletion of the HRP2 antigen existing in the DRC [63] and possibly to the prozone effect [63]. Conversely, false-positive results were also observed (in ~ 25% of positive RDT results) and can be explained by persistent HRP2 antigenaemia following resolved infections [64] or by cross-reaction with other diseases such as rheumatoid arthritis [65]. Overall, these findings suggest poor adequacy of HRP-2-based RDT for community surveys of *Plasmodium* spp. among adults. To effectively address infected adults in communities, the NMCP should take our results into account and consider updating its diagnostic policies which still rely primarily on HRP-2-based RDTs. Microscopy would have performed similarly to RDTs because nested PCR is known to detect approximately twice as many infections as microscopy in community surveys [13, 62]. Nested PCR methods have been acknowledged along quantitative PCR and nucleic acid sequence-based amplification for highly sensitive diagnosis of *Plasmodium* spp. in research settings [13]. These molecular methods can, in theory, detect parasitaemias as low as one gene copy per reaction or a single parasite in the blood sample [13]. Molecular detection tools should therefore be integrated into interventions and epidemiological measures to potentiate malaria control and elimination efforts targeting adults.

### Hosts' age profile and economic status may help optimize future interventions against *Plasmodium* spp. infections among asymptomatic adults

Addressing asymptomatic adults infected with *Plasmodium* spp. at community level in endemic areas requires a multi-pronged approach that goes beyond treating clinical cases. Strategies such as mass screening, mass or targeted drug administration or community engagement campaigns can be implemented. The logistical and financial burden of such mass interventions logically requires defining the population categories presenting the highest risks of infection to optimize the allocation of resources. In this regard, adults belonging to the “most economically disadvantaged” households were most exposed to *Plasmodium* spp. infections, whether *P. falciparum* or non-*falciparum* species. In accordance with this observation, existing evidence show that malaria affects the poorest categories of the population, being both a consequence and a cause of poverty and inequalities [3, 4]. Additionally, males were found with twice the risk of non-*falciparum* infections compared to females. *P. falciparum* infections were also more prevalent in males, although without reaching statistical significance. Evidence from school-age children and adults has long suggested this sex-based dimorphism in *P. vivax* and *P. falciparum* infections, often escribed to differences in exposure [66–68]. Likewise, sex differences in exposure or responses to diseases exist also for other pathogens [69, 70]. Beyond differential exposure, males have also been shown to clear more slowly asymptomatic *P. falciparum* infections than females, reflecting biological sex-based differences during host-parasite interactions and acquired immunity [71]. Hormonal differences, notably testosterone, would play a role in these sex-related immunological differences in *Plasmodium* spp. infections [72, 73]. However, further research is needed in this area to fully uncover the underlying mechanisms of these gender disparities. Furthermore, increasing the age of individuals by one year reduced the probability of infections by 1–2% among adults. *Plasmodium* spp. therefore disproportionately infected people under the age of 20 and gradually declined with age. Similarly, the age-related prevalence of *P. falciparum* infections peaks in people aged 10–14 years and gradually declines at older ages in the Congolese population [28]. Anti-parasite immunity acquisition in adulthood, following progressive maturation of the immune system (as opposed to anti-disease immunity acquired from childhood with repeated exposures), may partially explain this age-exposure relationship [10, 11]. Besides potential differences in host-parasite interactions and acquisition of immunity, the risk trends observed in this work may also be explained by differences in exposure due to behavioral factors such as occupational activities (e.g. example, school, agriculture, or night work), drug use and compliance with preventive measures [12]. Overall, categories of adult with higher *Plasmodium* spp. infection risks (e.g., the most economically disadvantaged, men, young adults) were identified, setting priorities to design tailored interventions to reduce transmission and ultimately eradicate malaria from the study regions and possibly elsewhere in the DRC.

### Study limitations

Several limitations of the present study should be considered when interpreting our findings. First, the community-based survey captured only adults from a specific location within a vast country. Children not included in the present study, who may also develop significant asymptomatic infections, should be considered in future investigations [20, 23]. Therefore, extrapolating our findings to any other context requires caution. Second, additional caution should be taken with the molecular methods used in this investigation, which may result in excessive positivity due to the persistence of parasite DNA left over from past infections [74]. Furthermore, conventional microscopy that could have provided information on blood parasite density, which is an important correlate of acquired immunity and transmission dynamics in the population, is sorely lacking [13]. Finally, this study is based in part on interviews and self-assessments which may involve significant information and memory biases.

## Conclusion

Notwithstanding the above limitations, this study has the merit of drawing attention to *Plasmodium* infections in adults, an often-neglected aspect of malaria epidemiology in endemic countries such as the DRC. Obtained results show that asymptomatic adults are broadly infected with *Plasmodium* spp. While *P. falciparum* remains the main species infecting adults, a surprisingly high contribution of non-*falciparum* species was noted, notably with *P. malariae* and *P. ovale*. Infected adults thus provide a large reservoir of *Plasmodium* spp. transmission, significant diagnostic challenges, complex malaria control efforts, and impact on individual health. This underscores the need for increased awareness of Plasmodium spp. infected adults and non-falciparum malaria in the study area. Effective interventions tailored to the population studied can be designed by prioritizing the socio-economic categories identified as most relevant in this survey (i.e. age, gender, and economic level). Molecular methods rather than common RDTs should be considered for diagnostic goals during various interventions targeting infected adults. Further research is needed to expand knowledge on *Plasmodium* spp. infecting adults and to establish programmatic and effective elimination strategies.

### Supplementary Information

**Additional file 1: Figure S1.** Surveys assessing P. falciparum infections across Africa. These maps display prevalence estimates of P. falciparum infections through the entire Africa (A) and through the DRC (B) based on the pulled data sources provided by the Malaria Atlas Project (MAP) (https://malariaatlas.org/pf-pv-cubes-2019/). Color palettes reflect the relative prevalence predicted at each site. The Lukelenge health zone where the current survey was conducted is indicated. **Table S1.** Baseline socio-demographic characteristics of the study population. **Table S2.** Self-reported history of putative clinical malaria episodes and use of preventive measures in the study population. **Table S3.** Qualitative agreement between assays for the detection of P. falciparum malaria in the study population. **Table S4.** Qualitative agreement between assays for the detection of non-falciparum malaria in the study population

**Additional file 2.** Survey Questionnaire

## Data Availability

All data generated during this study are included in the published article and its additional file.

## Abbreviations

DRC: Democratic Republic of Congo
RDT: Rapid diagnostic test
NMCP: National Malaria Control Program
LLIN: Long-lasting insecticide net
MAP: Malaria Atlas Project
PCR: Polymerase chain reaction
PCA: Principal component analysis
HRP-2: The *Plasmodium falciparum* histidine rich protein 2 antigen
COX3: Cytochrome c oxidase III
AIC: Akaike Information Criterion
IQR: Interquartile interval
AMED: The Japan Agency for Medical Research and Development
JSPS: The Japan Society for the Promotion of Science
