# Supplementary material for: Malaria infection among adults residing in a highly endemic region from the Democratic Republic of the Congo
Source: Malar J. 2024 Mar 18;23:82. doi: 10.1186/s12936-024-04881-7 (PMC10946143; doi:10.1186/s12936-024-04881-7)
Supplement: Supplementary file 1 — Additional file 1: Figure S1. Surveys assessing P. falciparum infections across Africa. These maps display prevalence estimates of P. falciparum infections through the entire Africa (A) and through the DRC (B) based on the pulled data sources provided by the Malaria Atlas Project (MAP) (https://malariaatlas.org/pf-pv-cubes-2019/). Color palettes reflect the relative prevalence predicted at each site. The Lukelenge health zone where the current survey was conducted is indicated. Table S1. Baseline socio-demographic characteristics of the study population. Table S2. Self-reported history of putative clinical malaria episodes and use of preventive measures in the study population. Table S3. Qualitative agreement between assays for the detection of P. falciparum malaria in the study population. Table S4. Qualitative agreement between assays for the detection of non-falciparum malaria in the study population [file 12936_2024_4881_MOESM1_ESM.pdf]

Supplementary file

Malaria infection among adults residing in a highly endemic region from the Democratic Republic of the Congo

Nadine Kayiba Kalenda*, Yuko Nitahara*, Evariste Tshibangu-Kabamba*, Denis Mbuyi Kalambayi, Augustin Kabongo-Tshibaka, Nestor Kalala Tshituka, Barthélemy Tshiebue Mukenga, Katherine-Sofia Candray-Medina, Natsuko Kaku, Yu Nakagama, Niko Speybroeck, Dieudonné Mumba Ngoyi, Ghislain Disashi Tumba, Akira Kaneko, Yasutoshi Kido[#]

[#] Correspondance: kidoyasu@omu.ac.jp

(*) Contributed equally

Contents

Contents 1

Figure S1. Surveys assessing *P. falciparum* infections across Africa 2

Table S1. Baseline socio-demographic characteristics of the study population 3

Table S2. Self-reported history of putative clinical malaria episodes and use of preventive measures in the study population 4

Table S3. Qualitative agreement between assays for the detection of *P. falciparum* malaria in the study population 5

Table S4. Qualitative agreement between assays for the detection of non-*falciparum* malaria in the study population 5

A

B

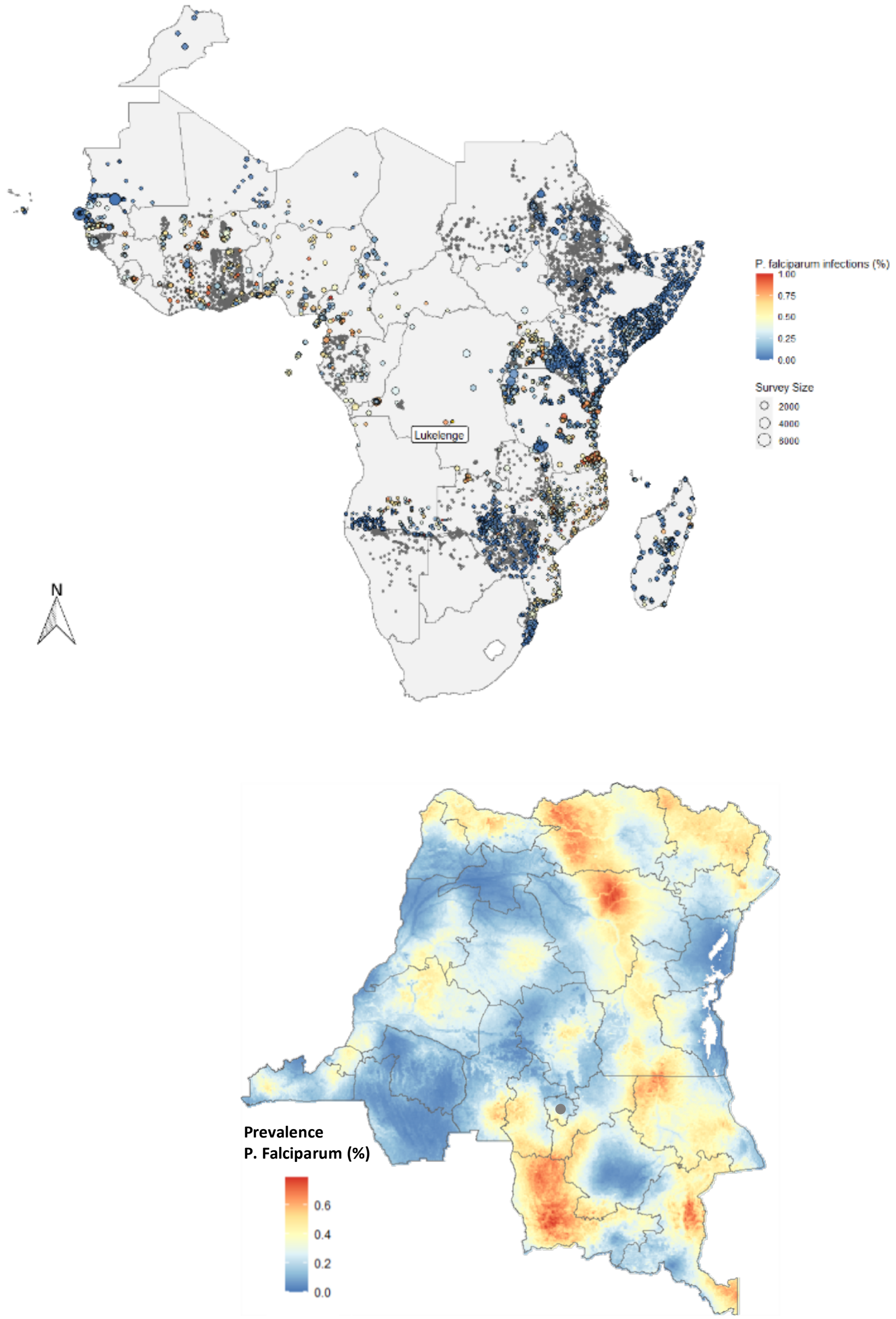

Figure S1. Surveys assessing *P. falciparum* infections across Africa.

These maps display prevalence estimates of *P. falciparum* infections through the entire Africa (**A**) and through the DRC (**B**) based on the pulled data sources provided by the Malaria Atlas Project (MAP) (https://malariaatlas.org/pf-pv-cubes-2019/). Color palettes reflect the relative prevalence predicted at each site. The Lukelenge health zone where the current survey was conducted is indicated.

Table S1. Baseline socio-demographic characteristics of the study population

Variable		PCR testing for *Plasmodium* spp.			p-value
		Positive	Negative	All	
		n or median (% or IQR)	n or median (% or IQR)	n or median (% or IQR)	
Age	Years	26 (23)	33 (31)	29 (25)	0.002
Gender	Female	146 (57.3)	106 (64.2)	252 (60.0)	0.153
	Male	109 (42.7)	59 (35.8)	168 (40.0)	
Marital status	Single	78 (30.6)	51 (30.9)	129 (30.7)	1,000
	Married, widower/widow, or divorced	177 (69.4)	114 (69.1)	291 (69.3)	
Religion	Christianity	247 (96.9)	158 (95.8)	405 (96.4)	0.551
	Other	8 (3.1)	7 (4.2)	15 (3.6)	
Education level	Elementary education level or below	53 (20.8)	42 (25.5)	95 (22.6)	0.264
	Higher education	202 (79.2)	123 (74.5)	325 (77.4)	
Profession	Employment	132 (51.8)	93 (56.4)	225 (53.6)	0.356
	Unemployment	123 (48.2)	72 (43.6)	195 (46.4)	
Crowding in households	No. of residents per sleeping room	2 (1.5)	2 (1.2)	2 (1.3)	0.272
Duration of residence in the study area	No. of years	4 (11)	4 (12)	4 (11)	0.322
Socio-economic category of the household (n=418)	'Most economically disadvantaged' (Q1)	194 (76.1)	87 (52.7)	279 (66.7)	<0.001
	'Less economically disadvantaged' (Q2)	61 (23.9)	78 (47.3)	139 (33.3)	
Main source for drinking water in the household	Tape water	253 (99.2)	163 (98.8)	416 (99.0)	0.647
	Water well or river	2 (0.8)	2 (1.2)	4 (1.0)	
Type of toilet facility for the household	Private toilets	35 (13.7)	26 (15.8)	61 (14.5)	0.564
	Community toilets	220 (86.3)	139 (84.2)	359 (85.5)	
Status of personal hand hygiene	Not optimal	230 (90.2)	150 (90.9)	380 (90.5)	0.808
	Optimal	25 (9.8)	15 (9.1)	40 (9.5)	

Table S2. Self-reported history of putative clinical malaria episodes and use of preventive measures in the study population

Variable		PCR testing for *Plasmodium* spp.			p-value
		Positive	Negative	All	
		n or median (% or IQR)	n or median (% or IQR)	n or median (% or IQR)	
Frequency of putative clinical malaria episodes in the past six months	No. of episodes	3 (3)	3 (4)	3 (3)	0.190
Estimated time past since the last malaria episode	No. of months	1 (2.5)	1 (2.7)	1 (2.6)	0.190
Putative acute malaria episode (fever or chill within 48 hours)	Yes	0 (0.0)	0 (0.0)	0 (0.0)	-
	No	253 (100.0)	167 (100.0)	420 (100.0)	
History of a clinical malaria episode in the last 3 months	Yes	204 (80)	135 (81.8)	339 (80.7)	0.645
	No	51 (20)	30 (18.2)	81 (19.3)	
Diagnostic confirmation of the most recent malaria episode	Unknown	49 (19.2)	29 (17.6)	78 (18.6)	0.766
	No	116 (45.5)	81 (49.1)	197 (46.9)	
	Yes	90 (35.3)	55 (33.3)	145 (34.5)	
Antimalarial drugs uptake at the most recent malaria episode	Unknown	53 (20.8)	30 (18.2)	83 (19.8)	0.400
	No	0 (0)	1 (0.6)	1 (0.2)	
	Yes	202 (79.2)	134 (81.2)	336 (80.0)	
Recent history of indoor insecticide spraying (in the last month)	Yes	1 (0.4)	6 (3.6)	7 (1.7)	0.016
	No	254 (99.6)	159 (96.4)	413 (98.3)	
Possession of long-lasting insecticidal nets (LLINs) in the household	No	146 (57.3)	94 (57)	240 (57.1)	1.000
	Yes	109 (42.7)	71 (43)	180 (42.9)	
No. of LLINs existing in the household	No. of LLINs	0 (1)	0 (1)	0 (1)	0.871
Use of LLINs at night (in the last month)	Yes	99 (38.8)	65 (39.4)	164 (39.0)	0.919
	No	156 (61.2)	100 (60.6)	256 (61.0)	
Applying environmental and hygiene measures to control malaria in or around the house	No	239 (93.7)	151 (91.5)	390 (92.9)	0.440
	Yes	16 (6.3)	14 (8.5)	30 (7.1)	
HRP2-based RDT outcome	Invalid	8 (3.1)	8 (4.8)	16 (3.8)	<0.001
	Negative	137 (53.8)	124 (75.2)	261 (62.1)	
	Positive	110 (43.1)	33 (20.0)	143 (34.0)	

Table S3. Qualitative agreement between assays for the detection of *P. falciparum* malaria in the study population

Detection method*		COX3 nested PCR for *P. falciparum*		Kappa [95%CI]	p-value
		Positive	Negative		
		n (%)	n (%)		
RDT	Positive	103 (72.0)	40 (28.0)	0.269 [0.176; 0.362]	<0.001
	Negative	110 (42.1)	151 (57.9)		
**Total**		**213 (52.7)**	**191 (47.3)**		

(*) Excluding 16 participants with an invalid RDT outcome

Table S4. Qualitative agreement between assays for the detection of non-*falciparum* malaria in the study population

Detection method*		COX3 nested PCR for non-*falciparum* species		Kappa [95%CI]	p-value
		Positive	Negative		
		n (%)	n (%)		
RDT	Positive	49 (34.3)	94 (65.7)	0.128 [0.018; 0.237]	0.013
	Negative	58 (22.3)	203 (77.7)		
**Total**		**107 (26.5)**	**297 (73.5)**		

(*) Excluding 16 participants with an invalid RDT outcome
