# Supplementary material for: Malaria infection among adults residing in a highly endemic region from the Democratic Republic of the Congo
Source: Malar J. 2024 Mar 18;23:82. doi: 10.1186/s12936-024-04881-7 (PMC10946143; doi:10.1186/s12936-024-04881-7)
Supplement: Supplementary file 2 — Additional file 2. Survey Questionnaire [file 12936_2024_4881_MOESM2_ESM.pdf]

SURVEY QUESTIONNAIRE

ID No : …
Date : …. / …./ ….
Site :
Investigator :

1. Socio-demographic information on the participant

Q1. What is your gender? R/ Female ☐ Male ☐ I prefer not to comment ☐
Q2. What is your date of birth? R/ .... / …. / …. Or estimated age: .... years (when failing to get the precise date of birth)
Q3. What is your marital Status? R/ Single ☐ Married ☐ Widower ☐ Divorce ☐
Q4. What is your home address? R/ No. …. Street ………………
Q5. What is your education level? R/ None ☐ Primary ☐ Secondary ☐ College/University ☐
Q6. What is your main professional/occupational activity?
R/ Salaried ☐ Craftsmanship ☐ Unemployment ☐ Schooling ☐ Other ☐
If "Other", please specify here: ….
Q7. What is your religion?
R/ Christianity ☐ Animism ☐ Islam ☐ Other ☐
If "Other", please specify here: ….

2. Socio-economic status of the participant's household

Q6. How long have you lived in this area ? R/ ….. years ….. months
Q7. What is the main financial source of your household? R/ …..
Q8. How much do you estimate your household monthly income to be? R/ …….. USD
Q9. What is your residential status? R/ Homeowner ☐ Tenant ☐ Other ☐
If "Other", please specify here: ….
Q10. How many members do you have in your household (people residing with you under the same roof)? R/ ….
Q11. How many rooms do you use for sleeping at night in your house? R/ ….
Q12. What are the main materials that make up the roof of your house?
R/ Sheet metal ☐ Straw ☐ Other ☐ If "Other", please specify here: ….
Q13. What are the main materials that make up the walls of your house?
R/ Cement block ☐ Baked bricks ☐ Clods of earth ☐ Stone with mud
Stone with lime or cement ☐ Mats/ bamboo/ thatch ☐
Q14. What are the main materials that make up the floor in your house?
R/ Flat stones ☐ Cement ☐ Clay ☐ Tile plate ☐

3. Symptoms, medical history, and malaria prevention

Q15. Have you suffered from malaria in the last six months? R/ Yes ☐ No ☐
If "Yes",
How many times? ….
Q16. When was your last episode of malaria (which month?)? R/ ….

Q17. Have you been tested to confirm malaria? Yes ☐ No ☐
Q18. Did you take a treatment for malaria? Yes ☐ No ☐
Q19. How many mosquito bed nets do you have in your household? R/ ……
Q20. How many nights have you spent under a mosquito bed net in the last month? R/ ....
Q21. Did you spend last night under an insecticide-treated mosquito net? Yes ☐ No ☐
Q22. Has there been any spraying of insecticide products in your home during the past month?
Yes ☐ No ☐
Q23. Has there been any environmental and hygiene measures applied to control malaria in or around your house during the past month?
Yes ☐ No ☐
Q24. Have you had a fever or chills in the past 3 days?
Yes ☐ No ☐
If "Yes", when was the last episode of fever or chills? .....
Q25. What symptoms did you have specifically during these 3 days?
R/ ….
….
….
Q18. Did you take a treatment for malaria in the last week? Yes ☐ No ☐
Q26. What is the current body temperature? R/ ….. ºC
(Please measure this with the thermometer provided)

4. Malaria testing results

Q27. HRP2-TDR results Positive ☐ Negative ☐ Invalid ☐
Q28. *Plasmodium* spp. PCR Positive ☐ Negative ☐
If positive, Pf ☐ Pv ☐ Pm ☐ Po ☐
